# Greenhouse gas emissions from tropical forest degradation: an underestimated source

**DOI:** 10.1186/s13021-017-0072-2

**Published:** 2017-02-14

**Authors:** Timothy R. H. Pearson, Sandra Brown, Lara Murray, Gabriel Sidman

**Affiliations:** Winrock International, 2121 Crystal Drive, Suite 500, Arlington, VA 22101 USA

**Keywords:** Carbon stock, Deforestation, Forest fire, Woodfuel, REDD+, Timber harvest

## Abstract

**Background:**

The degradation of forests in developing countries, particularly those within tropical and subtropical latitudes, is perceived to be an important contributor to global greenhouse gas emissions. However, the impacts of forest degradation are understudied and poorly understood, largely because international emission reduction programs have focused on deforestation, which is easier to detect and thus more readily monitored. To better understand and seize opportunities for addressing climate change it will be essential to improve knowledge of greenhouse gas emissions from forest degradation.

**Results:**

Here we provide a consistent estimation of forest degradation emissions between 2005 and 2010 across 74 developing countries covering 2.2 billion hectares of forests. We estimated annual emissions of 2.1 billion tons of carbon dioxide, of which 53% were derived from timber harvest, 30% from woodfuel harvest and 17% from forest fire. These percentages differed by region: timber harvest was as high as 69% in South and Central America and just 31% in Africa; woodfuel harvest was 35% in Asia, and just 10% in South and Central America; and fire ranged from 33% in Africa to only 5% in Asia. Of the total emissions from deforestation and forest degradation, forest degradation accounted for 25%. In 28 of the 74 countries, emissions from forest degradation exceeded those from deforestation.

**Conclusions:**

The results of this study clearly demonstrate the importance of accounting greenhouse gases from forest degradation by human activities. The scale of emissions presented indicates that the exclusion of forest degradation from national and international GHG accounting is distorting. This work helps identify where emissions are likely significant, but policy developments are needed to guide when and how accounting should be undertaken. Furthermore, ongoing research is needed to create and enhance cost-effective accounting approaches.

## Background

The degradation of forests in developing countries, particularly those within tropical and subtropical latitudes, is perceived to be an important contributor both to global greenhouse gas emissions and to development. Its impacts are understudied and poorly understood, and present a major challenge for national-level carbon inventories [7] and for addressing diminishing biodiversity [5]. International emission reduction programs (especially reducing emissions from deforestation and degradation, conservation of forest carbon stocks, sustainable management of forests and enhancement of forest carbon stocks—REDD+) have focused mostly on deforestation, which is easier to detect and thus more readily measured and monitored than forest degradation [13]. A key challenge for measuring and monitoring forest degradation is that it is difficult to detect using commonly-used remote sensing products, such as Landsat. Instead, much higher resolution imagery is needed to identify the more subtle changes in forest cover typical of forest degradation activity. The World Bank, a major REDD+ investor/donor, established a Carbon Fund [29] with a methodological framework that requires emissions from forest degradation to be accounted where ‘significant’, which is defined as more than 10% of ‘forest-related emissions’. Yet it is unclear how to quantify and meaningfully demonstrate “significance”, or how to account for emissions cost-effectively when significant.

Forest degradation occurs when there is a direct, human-induced decrease in carbon stocks in forests resulting from a loss of canopy cover that is insufficient to be classed as deforestation [11, 17]. Moreover, the decrease in carbon stocks should be persistent, although the duration of this persistence has not been defined. Common drivers of forest degradation include timber harvesting (legal and illegal), fuel wood collection, non-stand replacing fires, and animal grazing in the forest (preventing forest regeneration) [11].

A handful of studies have attempted to assess and quantify emissions from human-driven forest degradation, including an assessment of the importance of drivers of forest degradation made by Hosonuma et al. [14]. This study was based on data only for the area of forest disturbed in 46 tropical and sub-tropical countries. Of the total area of disturbed forests in these countries, they found that 51% of the disturbed area was caused by timber harvesting, 31% by woodfuel harvest, 9% by fires, and 7% by grazing. While timber harvest was the most significant activity in South and Central America and Asia, woodfuel was the largest activity by proportion (48%) in Africa. These estimates only included a subset of tropical and subtropical countries; were not produced through an independent and consistent assessment; and offered no quantitative information on the magnitude of the greenhouse gas emissions and how they compare to those from deforestation.

Another assessment of the emissions from forest degradation in the tropics conducted by Houghton [15] was based on his bookkeeping model for the period 1990–2010. He estimated that the average annual *net* emissions from harvesting of timber and woodfuel (with the exclusion of the re-clearing of forest fallow within the shifting cultivation cycle) just 10% of the summed emissions from deforestation and degradation, with degradation emissions dominated by timber harvest with marginal emissions from woodfuel, and no emission from fires. Given the exclusion of other key causes of forest degradation, this study is incomplete and lacks consistency.

Recent work by Pearson et al. [24] focused on the perceived key cause of forest degradation: timber harvest and associated infrastructure (skid trails and logging roads). They showed that for nine major tropical timber producing countries, emissions from logging were on average equivalent to about 12% of those from deforestation. For those nine countries with relatively low emissions from deforestation, emissions from logging were found to be equivalent to half or more of those from deforestation, whereas for countries with the highest emissions from deforestation, emissions from logging were equivalent to <10% of those from deforestation. These estimates are supported by the work of Asner and others in the Brazilian Amazon. Asner et al. [3] estimated logged areas ranged from 60 to 123% of previously reported deforestation areas. Huang and Asner [16] estimated that the inclusion of timber harvest elevated emissions by 15–19% over the emissions from deforestation alone.

Collection of traditional woodfuel (firewood and charcoal) for cooking and heating is common throughout the tropics, and can lead to forest degradation where removals exceed regrowth. Where annual harvest of woodfuel exceeds the forest’s incremental growth in biomass, it is considered to be unsustainable, and leads to a decline of woody biomass and to net carbon emissions [4]. Bailis et al. [4] estimated that 27–34% of woodfuel harvest was unsustainable, particularly in East Africa and South Asia, and thus leads to significant forest degradation.

Fire is an important cause of forest disturbance and is commonly used to manage forest lands in the tropics and subtropics [28]. Fire is often used to transform forest, e.g. into croplands, but this is a land-use change and so is considered to be deforestation rather than forest degradation. When fires in forests are not associated with an intentional conversion for a land-use change, this is considered to be forest degradation. The work by van der Werf et al. [28] included an analysis for tropical latitudes that partitioned the forest fires into two classes: non-deforestation fires (i.e. forest degradation), and deforestation fires.

It is clear that no estimates of CO_2_ emissions currently exist that incorporate all major forms of forest degradation. Thus, a systematic, consistent calculation approach is needed to allow for an estimation of all significant emissions across all tropical and subtropical developing countries. Such improved knowledge on emissions from forest degradation would allow decision makers to understand the extent of forest degradation and what opportunities there are to reduce associated emissions. As such, the goals of our work were to: (1) provide a consistent estimate of CO_2_ emissions from the major causes of degradation in the tropical and subtropical forests of developing countries, and (2) compare the magnitude of the emissions caused by forest degradation and its sub-activities with those from deforestation in both absolute and relative terms. Results from such an analysis would provide guidance to national and international policy makers as to which forest lands to allocate resources so that national GHG emissions are reduced.

## Methods

**Fig. 1:**
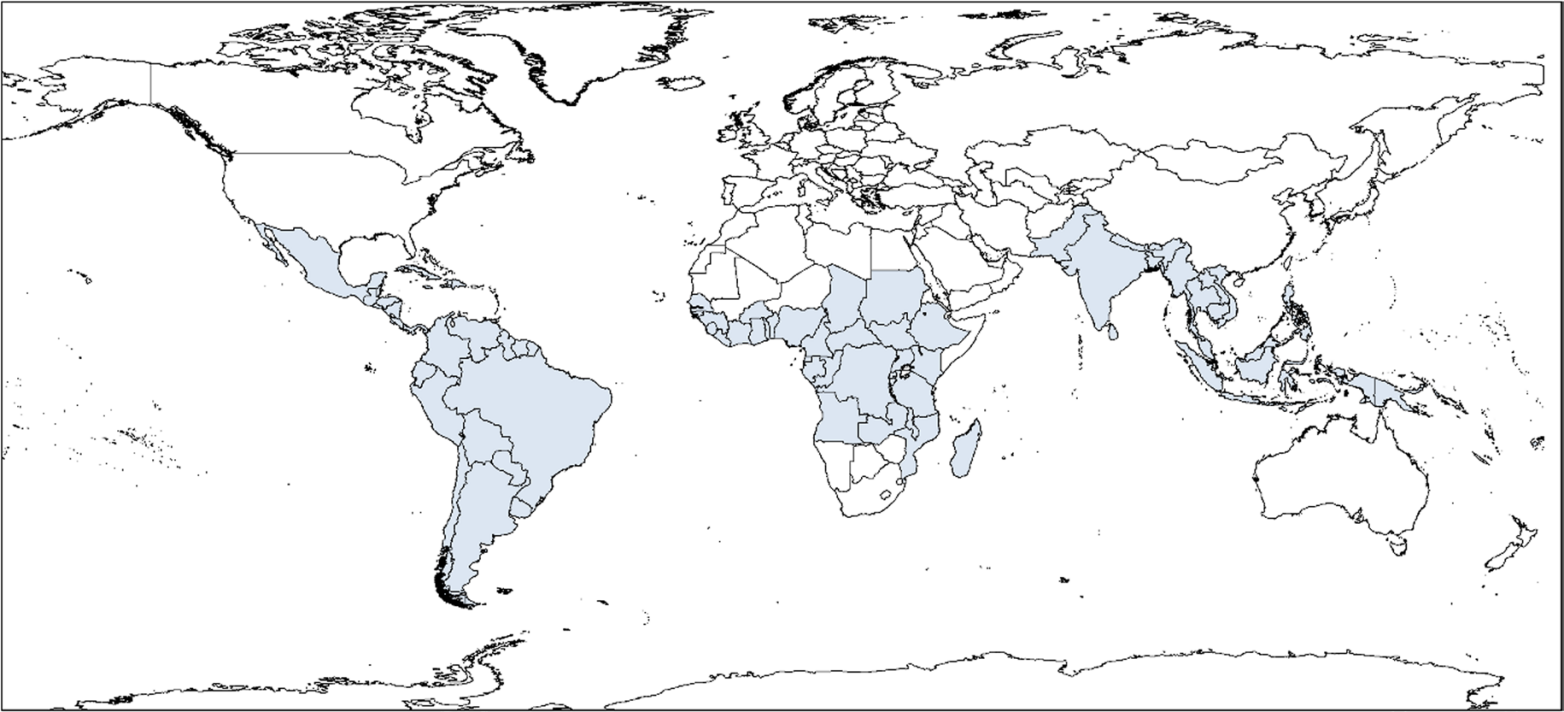
Map of included countries (shaded in *blue*)

Our analysis of emissions from forest degradation covers 74 developing countries located mostly in tropical and subtropical latitudes. These countries contain 4.7 billion hectares of land area and 2.2 billion hectares of forest (Fig. 1). The three main causes of forest degradation included in this analysis are:

- Selective timber harvest in native forests.
- Woodfuel harvest—where removals exceed regrowth of forest C stocks.
- Fire—wildfires that do not cause a change in land-use.

All estimates of emissions for each activity are of gross emissions, and do not take into account how persistent the degradation might be or any regrowth and forest recovery. All estimates are derived from global databases and the scales, carbon pools, and time frame for each activity are given in Table 1.

**Table 1:** Summary of activities, spatial scale, pools and time frame included in the analysis

Activity	Source	Spatial scale	Pools included	Time frame
Timber harvest	[24]	National	Above and belowground live biomass, harvested wood products	2005–2010
Wood fuel harvest	WISDOM Model	GADM Level 1	Above and belowground live biomass	2009
Fire	Global Fire Emissions Database [28]	50 km Summed to GADM Level 1	Above and belowground live biomass, dead wood, and litter	2005–2010
Deforestation	[12, 25]	Area—30 m Stocks—250 m Summed to GADM Level 1	Above and belowground live biomass, dead wood, litter, and soil carbon	2005–2010

*GADM* database of global administrative areas (http://www.gadm.org)

### Degradation

#### Selective timber harvest

The methodology described in Pearson et al. [24] was used to estimate total emissions from selective timber harvest. Emissions include those from (1) direct carbon loss of the extracted log (extracted log emissions—ELE); (2) the top and stump of the felled tree, plus trees incidentally killed or severely damaged surrounding the logging gap (logging damage factor—LDF); and (3) trees killed during the construction of logging infrastructure (logging infrastructure factor—LIF). This method combines these sources of emissions associated with selective timber harvesting to derive a single emission factor that is applied to the volume of timber extracted. The inverse of emissions is carbon stored therefore the calculation of emissions captures both all losses and the impact of carbon stored in long-term wood products (e.g., in furniture or buildings). Pearson et al. [24, Feldpausch et al. 9], and unpublished data from Ghana provide estimates of the emission factors for seven tropical forested countries based on field data collection as shown in Table 2. These factors were applied in this study based on what region the countries are best suited to represent, as shown in Table 2.

**Table 2:** Source of field data for development of timber harvesting emission factors (*ELE* extracted log emission, *LDF* logging damage factor, *LIF* logging infrastructure factor)

Region	ELE	LDF	LIF	Country
Central Africa	0.25	0.5	0.24	Republic of Congo
Rest of Africa	0.37	0.67	0.24	Ghana
Central America and Caribbean	0.28	1.26	0.27^a^	Belize
Andean countries^b^ (Bolivia, Colombia, Ecuador, Paraguay, Peru, Venezuela)	0.30	1.23	0.27^a^	Bolivia
Brazil	0.38	0.71	0.27^a^	Brazil
Guyana, Suriname, French Guyana	0.36	0.99	0.98	Guyana
Asia	0.25	0.57	0.67	Indonesia

All factors are in units of Mg C m^−3^

^a^The values for the LIF are from Feldpausch et al. [9]

^b^These countries are mostly Andean but grouped into once class

Average annual industrial roundwood production (IRP), a measure of the extracted volumes, for the period of 2005–2010 was obtained from the FAO Global Forest Resources Assessment database (FAOSTAT) [10], as well as the country reports submitted to the FAO as part of the Forest Resource Assessment (FRA) program. Because the reported IRP include volumes produced from native forests and forest plantations, the reported IRP was adjusted to ensure that only timber production from native forests was considered (to capture only emissions from selective logging). For the majority of timber-producing countries included in the analysis (representing 96% of the total IRP), country-specific harvest volumes from plantations for the 2005–2010 timeframe reported in Jürgensen et al. [20] were subtracted from the average total industrial roundwood production volume, as reported by FAOSTAT for the same time period. For countries not included in Jürgensen et al. [20], no adjustments were made, as we assumed that IRP from plantations (if they exist) were insignificant.

#### Woodfuel

Emissions from woodfuel were derived using the WISDOM model [4] that estimates the fraction of non-renewable biomass (NRB) in relation to supply and demand potential [4]. In the WISDOM model, woodfuel derived as a byproduct of deforestation activities was not included in order to avoid double-counting deforestation emissions. As the WISDOM model estimates only include the aboveground biomass pool, an expansion factor of 1.32 was applied to conservatively estimate the total biomass, based on the American Carbon Registry’s Energy efficiency measures in thermal applications of nonrenewable biomass methodology [2], based on the CDM-approved methodology AMS‐II.G, Version 05.0. This factor assumes that for every unit of biomass extracted from the forest, an additional 10% is left in the field from uncollected aboveground biomass. A further 20% is conservatively estimated to remain from root biomass.

#### Fire

The Global Fire Emissions Database (GFED; [28]) was used for estimates of emissions from forest fire. The GFED provides a global monthly layer with a cell size of 0.5 decimal degrees (approx. 50 × 50 km) of dry matter emissions that are classified into different sources and land cover types. Within the humid tropical forest biome, fire emissions from deforestation are decoupled from other emissions based on fire persistence (the length of time for which a fire burns in the same location). To avoid double-counting with deforestation emissions, only emissions from GFED-classified forest fires within latitudes 23° North and South (and not deforestation fires) were used in this degradation category. The GFED3 monthly layers from 2005–2010 were used for this study, and emissions estimates for only CO_2_ are reported here in order to be consistent with other degradation activities.

### Deforestation

Although there are several estimates of CO_2_ emissions from tropical deforestation published fairly recently (e.g. [1, 6, 13, 15, 26, 30]), these estimates were not used because they were not consistent with respect to carbon pools included, area of study, definition of forest, inclusion of other land-use changes, gross versus net emissions, and years covered. As one of our goals was to compare estimates of degradation emissions with those of deforestation, we believed it was important to estimate the emissions from deforestation in a manner consistent with our analysis of forest degradation (Table 1).

Emissions were obtained by multiplying the average forest carbon stocks for each administrative unit by the area of forest loss. We used the Hansen et al. [12] dataset, derived from Landsat 7 ETM+ satellite images, to determine the area of deforestation. Deforestation data was based on a canopy closure of 20% to ensure that deforestation in countries with more open, drier forests were well captured. Areas shown as loss (between 2005 and 2010) were considered to be deforested, and were summed across level-one subnational administrative units as defined by the GADM (Database of Global Administrative Areas; political boundaries reflecting states or districts).

Tropical peatswamp forests under threat for deforestation are overwhelmingly located in Indonesia and Malaysia (more than 56% of area), with the remainder generally located in areas where pressure for deforestation is very low including at high altitudes in the mountains of Africa, South America and Papua New Guinea [23]. A spatial layer of peat forest areas in Indonesia and Malaysia was created using information from the Harmonized World Soil Database (HWSD; FAO/IIASA/ISRIC/ISS-CAS/JRC. Harmonized world soil database [8]. FAO, Rome, Italy and IIASA, Laxenburg, Austria 2012). All soil units classified as histosols (a soil consisting primarily of organic materials and defined as having 40 cm or more of organic soil material in the upper 80 cm) were assumed to be peat soil in these two countries. All areas of deforestation according to the Hansen et al. [12] layer that occurred on peat in Indonesia and Malaysia were assumed to be deforestation of peatswamp forests, and the method to estimate soil emissions is given in Table 3. The emissions for non-peat soils use the soil C stock to 30 cm deep given in the HWSD and the IPCC [18] land-use change factors (Table 3).

**Table 3:** Source of data for calculating emissions from deforestation

Pool	Source
Aboveground live	Saatchi et al. biomass map ([25]; and unpublished update to 2011 increasing resolution from 500 to 250 m and adding additional ground data) Forest mask for year 2005 from Hansen et al. [12] to exclude non-forest biomass pixels
Belowground live	Equations from Mokany et al. [22]
Dead organic matter	Fraction of aboveground biomass [27]
Soil organic matter	Peat soil emissions—annual emission factor for drained organic soil applied for 10 years (5.3 t CO_2_ ha^−1^ year^−1^; [19]) Non-peat soil emissions: C stock in top 30 cm from HWSD database Land use change soil factors from IPCC [18]

Carbon stocks of the non-soil pools were derived as detailed in Table 3. Biomass was averaged across the subnational administrative units and carbon stocks from all pools were assumed to be committed to the atmosphere immediately at the time of deforestation. Emissions were obtained by multiplying the average forest carbon stocks for each administrative unit by the area of forest loss.

## Results

We estimated that total emissions from forest degradation were 2.1 Gt CO_2_e (Table 4) across the 74 countries assessed. Emissions associated with timber harvest accounted for more than half of the total degradation emissions (53%) followed by woodfuel (30%) and fire (17%). Emissions from forest degradation represented 25% of the estimated total emissions from deforestation plus forest degradation.

**Table 4:** Estimated annual emissions from deforestation and forest degradation and relative proportions

Activity	Annual emission (Gt CO_2_e year^−1^)	%
Degradation	2.06	25
Timber	1.09	53
Woodfuel	0.62	30
Fire	0.35	17
Deforestation	6.22	75

**Fig. 2:**
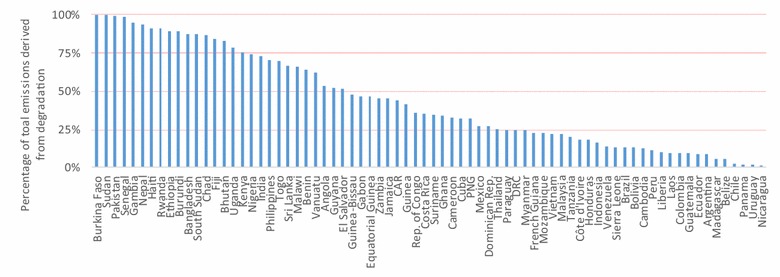
Proportion of total forest emissions from forest degradation for the 74 countries included in this study

Although emissions from forest degradation for all countries included in this study accounted for just a quarter of the total emissions (deforestation and forest degradation combined), for 28 of the 74 countries (38%), more than half of the total emissions were derived from forest degradation. Estimates of emissions from all sources of forest degradation were less than 10% in only 11 countries (Fig. 2; recall that where forest degradation is less than 10% of emissions from all sources, it may be omitted from accounting under the World Bank methodological framework for REDD+). The highest proportion of degradation emissions relative to total emissions (>75%) were found to occur in the more arid countries of South Asia and north and east Africa (Fig. 2).

**Fig. 3:**
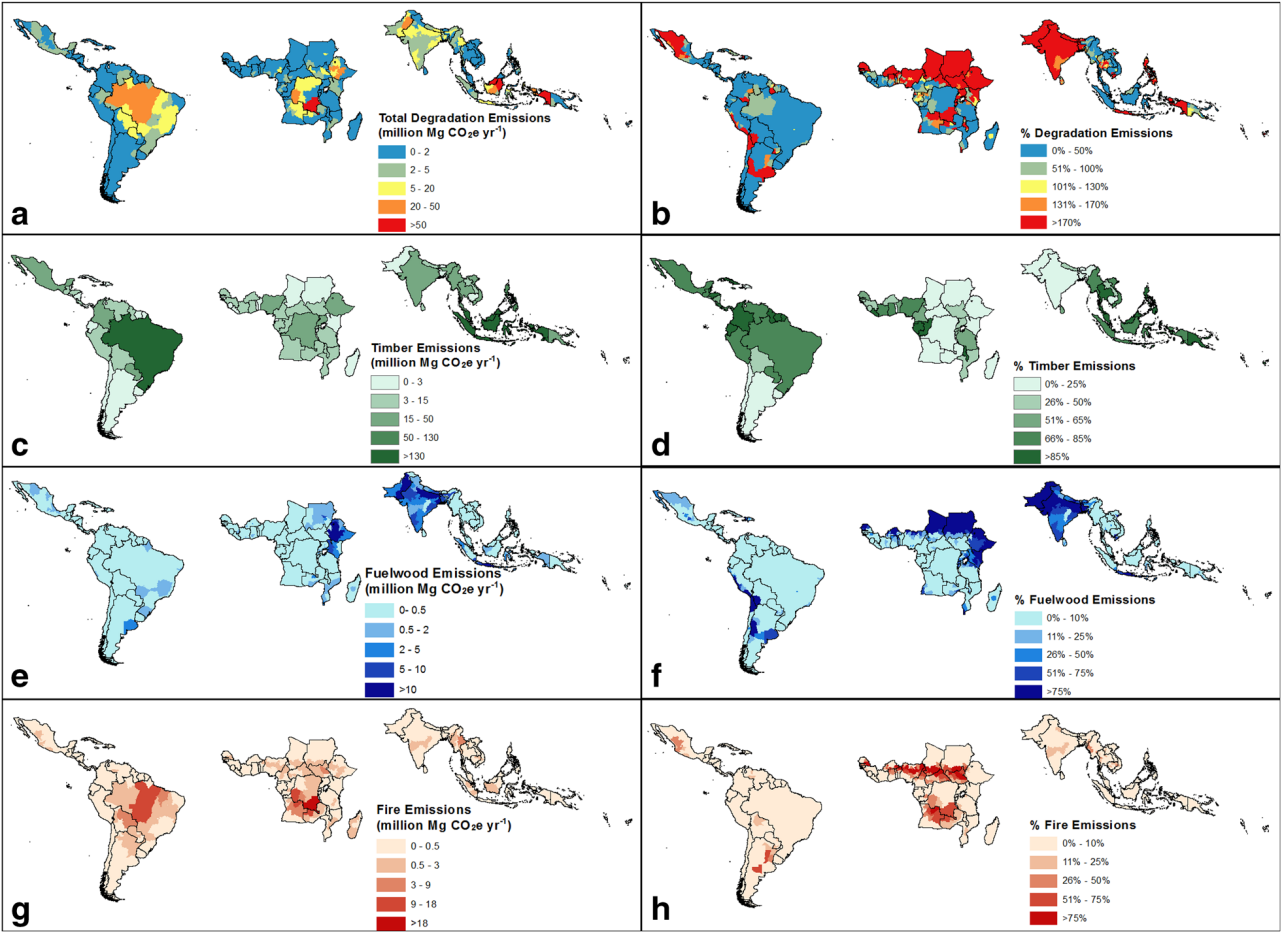
Spatial distribution of forest degradation emissions and percent of total forest emissions for: **a**, **b** total degradation emission by region within countries, **c**, **d** timber extraction emissions (only national level), **e**, **f** woodfuel emissions, and **g**, **h** fire emissions

The magnitude of total degradation emissions was highest in the largest forested countries, led by Brazil and Indonesia (Fig. 3a, b). Timber production was the largest source of degradation emissions for these countries (Fig. 3c, d). Woodfuel emissions were highest in South Asia, Indonesia and in east Africa. Notable emissions from fire occurred in DRC and parts of the Brazilian Amazon (Fig. 3g, h). However, proportionally, fire emissions were highest (about 75% or more of total emissions) for parts of Bolivia and Argentina in South America and for Central African countries (Fig. 3h).

**Fig. 4:**
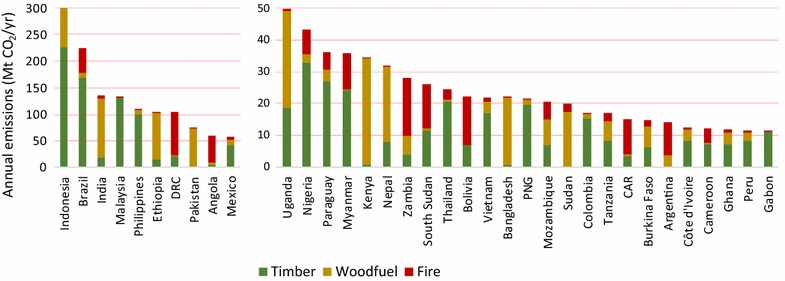
Annual forest degradation emissions disaggregated by cause for the 35 countries with the highest emissions

The 35 countries with the greatest forest degradation emissions are divided into two groups—the top 10 with emissions >50 Mt CO_2_ year^−1^ and the next 25 with emissions <50 Mt CO_2_ year^−1^—and are displayed with the varied proportion by source of emission (Fig. 4). The contribution of emissions by driver differs for these countries; timber harvest was the main cause for 5 of the top 10 countries, followed by woodfuel for three countries, and fire for the last two. For the countries in the second group, timber production was still the dominant cause for about half of them; and the dominant cause for remaining countries was equally divided into woodfuel and fire. Emissions from woodfuel are not correlated to the area of forest—several countries in Africa with relatively small areas of forest have high emissions from degradation due to woodfuel harvest. Emissions from woodfuel in general are relatively high in East Africa and South Asia, where it is a primary source of energy for cooking in not only in rural areas but also in urban areas (these two regions represent 71% of global woodfuel emissions; 439 Mt CO_2_). For the relatively more developed countries of South and Central America and the Caribbean, emissions caused by woodfuel harvests are insignificant. This is likely because alternate fuel sources are used and there is plenty of woodfuel available from timber harvesting offcuts.

**Fig. 5:**
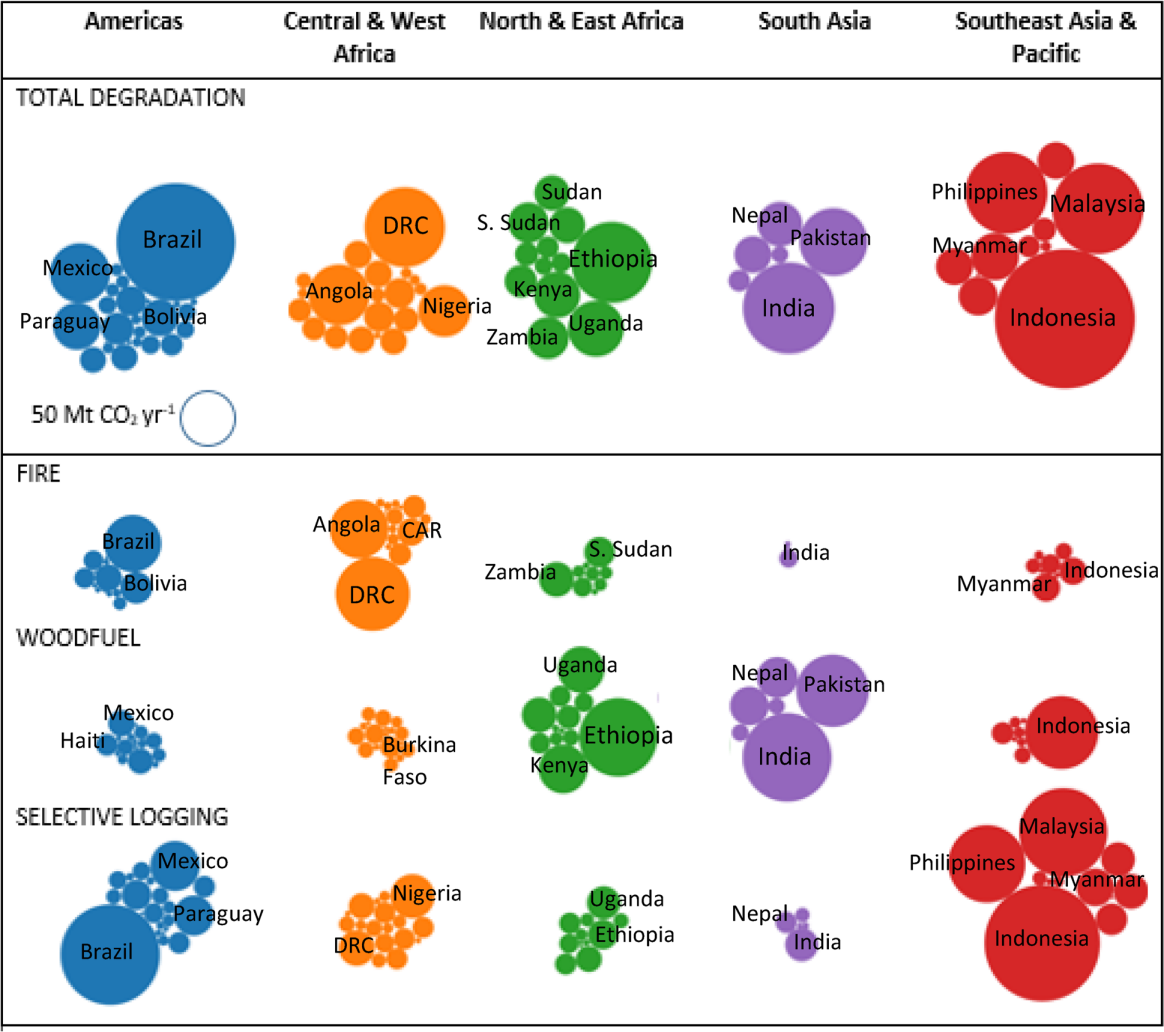
*Bubble charts* showing degradation emissions by region. The *size of the bubbles* represents the relative magnitude of annual emissions

This study reveals distinct patterns whereby dominant sources of emissions are split by region and continent (Fig. 5). South America and Southeast Asia contribute the most emissions from forest degradation (>51%), which can be attributed to their vast areas of high carbon stock forests. Forests in countries of Central America and the Caribbean as well as East Africa account for the least amount of degradation emissions (about 12%) due to their relatively small area of forests, many of which have low carbon stocks.

## Discussion

### Comparison of emissions from forest degradation

This study offers the first complete and consistent analysis of gross emissions from activities associated with the degradation of forest lands in developing countries in the tropical and subtropical latitudes. We estimated total forest degradation emissions of 2.1 Gt CO_2_e year^−1^, of which 53% was derived from timber harvest, 30% from woodfuel harvest, and 17% from forest fires.

Although Hosonuma et al. [14] did not quantify emissions, that study presented the proportion of total degradation resulting from each degradation activity (self-estimated by countries) for a subset of the countries included in our study area. Hosonuma et al. [14] estimated degradation emission sources as 51% from timber harvest, 31% from woodfuel and 9% from fires (compared to our results of 53, 30, 17%). Breaking down by continent, Hosonuma et al. found that timber harvest exceeded 70% in South and Central America and Asia, but were just over 30% in Africa; woodfuel was 48% in Africa but less than 20% in Asia, and less than 10% in South and Central America; while fire was less than 20% in South and Central America, less than 10% in Africa and less than 5% in Asia. Thus the findings of Hosonuma et al. are largely in agreement with the findings of this study (Table 5) and highlight that the harvesting of timber and woodfuel are the largest contributor of emissions associated with forest degradation.

**Table 5:** Proportion of total forest degradation emissions by degrading activity by region

	Timber (%)	Woodfuel (%)	Fire (%)
America	69	10	21
Africa	31	36	33
Asia	61	35	5

For another comparison we can specifically compare emissions from timber harvesting in the Brazilian Amazon. Huang and Asner [16] estimated annual gross emissions as 0.15–0.18 Gt CO_2_e year^−1^. Comparing just the nine Brazilian states that comprise the Amazon region, our study estimates emissions to be 0.28 Gt CO_2_e year^−1^, or more than 1.5 times higher than those reported by Huang and Asner. However, the Huang and Asner study explicitly stated that their estimate of gross annual emissions was likely to have substantially underestimated emissions due to the exclusion of areas that were deforested in subsequent years.

### Emissions from deforestation versus forest degradation

The estimate of gross deforestation emissions presented in this study (average annual for 2005–2010 is 6.22 Gt CO_2_) is included primarily to serve as a basis for consistent comparison with the estimates of degradation emissions. Recent published estimates of deforestation emissions [1, 6, 13, 15, 26, 30] have been smaller than our estimate, ranging from 2.3 to 4.2 Gt CO_2_ year^−1^. There are several reasons for the discrepancy between these estimates, including a focus on net rather than gross emissions, different time periods which will capture lower historical rates of deforestation—e.g. 2000–2005 [13] to 2001–2013 [30]—and different study areas. All of the estimates generally include only aboveground biomass carbon stocks in trees (except [13], which also included belowground biomass), yet our estimate includes all five IPCC carbon pools, including aboveground, belowground, dead wood, litter, soil, and peat. Belowground biomass of forests is about 20% or more of aboveground biomass and dead wood and litter will account for at least another 5% of aboveground biomass. Emissions from mineral soil due to cultivation generally account for another 20–25% of aboveground stocks. Taking all these factors into account, the emissions from the other studies could increase by as much as a factor of 1.5, or to a range of 3.5–6.3 Gt CO_2_ year^−1^, while still not including significant peat soil emissions in Indonesia and Malaysia. In light of all this, we conclude that our estimate of deforestation emissions is in line with other recently published estimates mentioned above.

Emissions from forest degradation are not an insignificant source of CO_2_ and account for 25% of the summed emissions from deforestation and forest degradation of 8.28 Gt CO_2_ year^−1^. In other words, degradation emissions are equivalent to about a third of those from deforestation. According to the World Bank’s Carbon Fund, if emissions from forest degradation are more than 10% of all forest-related emissions, they must be included and accounted for. As we have shown, emissions from all sources of forest degradation were less than 10% in only 11 out of the 74 countries, and thus all the remaining countries would need to include forest degradation in their accounting system. The guidelines, however, only give instructions on summed forest degradation but not on individual activities. For example, in Colombia summed degradation emissions were equal to 9% of total emissions, but all the emissions are from timber harvest and thus could be excluded under FCPF rules. In contrast, the summed degradation emissions in Peru were 11% but the timber harvest emissions comprised 8% of total degradation. While Peru’s emissions from timber degradation are less significant than in Colombia, since total degradation emissions make up more than 10%, Peru would be required to also account for fire and woodfuel even though they sum to just 3% of emissions. Thus, there is a need for policies that better articulate the inclusion and exclusion of activities rather than the summed forest degradation level.

### Significance of degradation emissions

**Fig. 6:**
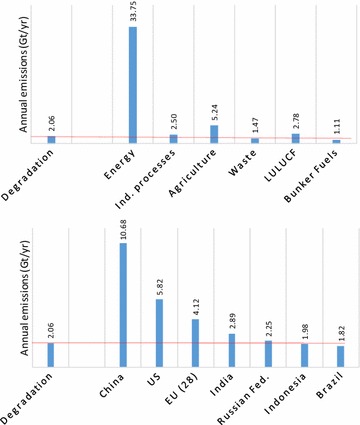
Estimated total greenhouse gas emissions from forest degradation relative to other emission sectors (*upper figure*) and relative to large emitting countries (*lower figure*). Values derived from WRI CAIT (http://cait.wri.org)

The consistent estimates of emissions produced in this study allow us to consider the significance of total emissions resulting from forest degradation. To better illustrate this significance, we directly compared our estimates with emissions by country and emission sector as listed by the WRI CAIT database (http://cait.wri.org) for 2010. According to this comparison, degradation emissions are only significantly exceeded by the energy and agriculture sectors (Fig. 6). On a country basis, total emissions from forest degradation exceed all but the seven highest emitting countries (Fig. 6).

### Uncertainties and omitted sources

The purpose of this analysis was to demonstrate the scale of emissions from forest degradation in a manner that is to the best of our knowledge consistent and accurate. This requires accurate information on extent of the type of forest degradation and the associated emissions. For selective logging, there was concern about the data used to estimate emissions, as it may have included timber volumes derived from plantations. However, steps were taken to ensure that our estimates of IRP capture extraction rates only from native forests. The logging emission factors were developed using data only from a limited number of countries yet have very small error bounds, and the emission sources considered have significant relationships with forest characteristics [24]. The fire analysis is spatially-specific and globally-consistent, and was designed to avoid double counting fire degradation emissions with fire emissions resulting from or associated with deforestation. The most uncertain emission source is woodfuel, given that the data are derived from a single year.

Estimates of emissions from timber harvest are likely to be underestimated due to the omission of illegal logging, assuming illegal logging is not included in national official statistics of IRP. It is important to acknowledge that research indicates that as much as 72% of logging is illegal in the Brazilian Amazon, 61% in Indonesia and 65% in Ghana [21].

Another omission is degradation through overgrazing. This source was included in Hosonuma et al. [14], who reported that this activity is responsible for 7% of the pantropical area of forest degradation (the least important form of degradation in the study). In addition, the impact of grazing is predominantly on regeneration, with damage to seedlings and saplings. The impact on forest carbon stocks is therefore small in the short term, though may be greater in later years as future generations of emergent trees are removed.

## Conclusions

Our estimates show annual forest degradation emissions of 2.1 billion tons of carbon dioxide across 74 developing countries. To further illustrate the significance of this number: it exceeds both the total emission from highway vehicles (1.7 billion tons of carbon dioxide equivalents per year; fueleconomy.gov accessed 1/27/17), and the total emissions from power generation in the USA (1.9 billion tons of carbon dioxide equivalents per year; eia.gov accessed 1/27/17).

Our study demonstrates that, almost without exception, forest degradation emissions are significant. Indeed, by our estimates 85% of the countries studied surpass the defined minimum threshold and would be required to estimate forest degradation emissions under World Bank requirements for participation in the Carbon Fund REDD+ program.

Yet emissions from forest degradation are overlooked and not accounted in any complete or systematic way. It is imperative that this source of greenhouse gas emissions be better understood so that strategies that tap into the mitigation potential of addressing them may be developed. These strategies might in turn also offer significant economic and development opportunities.

This paper serves as a starting point to demonstrate the importance of forest degradation as a source of greenhouse gases, and to show where emissions are most significant—and thus where interventions may have the greatest impact.

## Abbreviations

CO_2_: carbon dioxide
ELE: extracted log emissions
FAO: Food and Agriculture Organization
GHG: greenhouse gases
HWP: harvested wood products
IPCC: Intergovernmental Panel on Climate Change
IRP: industrial roundwood production
LDF: logging damage factor
LIF: logging infrastructure factor
REDD+: reducing emissions from deforestation and degradation, conservation of forest carbon stocks, sustainable management of forests and enhancement of forest carbon stocks
NRB: non-renewable biomass
